# Effects of COVID-19 infection in patients with autoimmune pulmonary alveolar proteinosis: a single-center study

**DOI:** 10.1186/s13023-023-02950-9

**Published:** 2023-11-11

**Authors:** Chuanxin Duan, Wangji Zhou, Miaoyan Zhang, Chongsheng Cheng, Wenshuai Xu, Jinrong Dai, Shuzhen Meng, Keqi Chen, Yang Zhao, Song Liu, Shao-Ting Wang, Yanli Yang, Kai-Feng Xu, Xinlun Tian

**Affiliations:** 1 Department of Pulmonary and Critical Care Medicine, State Key Laboratory of Complex Severe and Rare Diseases, Peking Union Medical College Hospital, Chinese Academy of Medical Sciences, Peking Union Medical College, #1 Shuaifuyuan, Wangfujing, Beijing, 100730 China; 2 Center of Medical Research, Peking Union Medical College Hospital, Chinese Academy of Medical Sciences, Peking Union Medical College, Beijing, China

**Keywords:** Autoimmune pulmonary alveolar proteinosis, COVID-19, Oxygen saturation

## Abstract

**Background:**

Autoimmune pulmonary alveolar proteinosis (aPAP) is a rare interstitial lung disease. COVID-19 is associated with worse prognosis in previous lung diseases patients. But the prognosis of aPAP patients after infection with COVID-19 is unclear. In December 2022, China experienced a large-scale outbreak of Omicron variant of the SARS-CoV-2. In this study, we aim to explore the clinical outcomes of aPAP patients infected with COVID-19.

**Results:**

A total of 39 aPAP patients were included in this study. 30.77% patients had a decrease in oxygen saturation after COVID-19 infection. We compared the two groups of patients with or without decreased oxygen saturation after COVID-19 infection and found that patients who had previous oxygen therapy (decreased oxygen saturation vs. non decreased oxygen saturation: 6/12 vs. 4/27, *P* = 0.043), with lower baseline arterial oxygen partial pressure (74.50 ± 13.61 mmHg vs. 86.49 ± 11.92 mmHg, *P* = 0.009), lower baseline DLCO/VA% [77.0 (74.3, 93.6) % vs. 89.5 (78.2, 97.4) %, *P* = 0.036], shorter baseline 6MWD [464 (406, 538) m vs. 532 (470, 575) m, *P* = 0.028], higher disease severity score (*P* = 0.017), were more likely to have decreased oxygen saturation after COVID-19 infection.

**Conclusion:**

aPAP patients with poor baseline respiration have a higher probability of hypoxia after COVID-19 infection, but fatal events were rare.

**Supplementary Information:**

The online version contains supplementary material available at 10.1186/s13023-023-02950-9.

## Background

Pulmonary alveolar proteinosis (PAP) is a rare interstitial lung disease, with a prevalence of at least 7 per million people in large population studies. There are three types of PAP, namely primary, congenital and secondary PAP. Primary PAP can be divided into autoimmune PAP and hereditary PAP [1]. Autoimmune pulmonary alveolar proteinosis (aPAP) is the most common type of PAP, originally known as idiopathic PAP or acquired PAP, and more than 90% of patients with PAP are of this type [2]. The anti granulocyte‑macrophage colony‑stimulating factor (GM‑CSF) antibody in the blood of patients with aPAP blocked the signaling of GM‑CSF, resulting in the dysfunction of alveolar macrophages in scavenging surfactant. aPAP can be diagnosed by detecting the presence of anti GM-CSF antibody in serum. For aPAP patients with treatment indications, inhalation of GM-CSF or whole lung lavage (WLL) can be used for treatment [3].

SARS-CoV-2, a new infection that causes COVID-19, is associated with worse prognosis in individuals with previous lung diseases [4]. Therefore, it is not difficult to speculate that patients with aPAP may have more serious complications and worse prognosis after infection with SARS-CoV-2. A European retrospective cohort study reported that the prevalence of COVID-19 was similar in the PAP population compared with the general population, but both the rates of hospitalizations and mortality were higher [5]. Meanwhile, some case reports have found that inhaling GM-CSF is feasible for PAP patients with COVID-19, while WLL therapy is controversial [6–8]. In December 2022, China experienced a large-scale outbreak of Omicron variant of the SARS-CoV-2. In this context, our research aims to explore the clinical outcomes of aPAP patients infected with SARS-CoV-2.

## Methods

### Study population

The patients included in the study were all from the Chinese PAP registry study follow-up cohort, while all were patients with aPAP diagnosed at Peking Union Medical College Hospital (PUMCH). The inclusion criteria included: (1) patients with a clinical diagnosis of PAP by high-resolution computed tomography (HRCT) and further pathologically reported to have positive staining for proteinaceous material periodic Acid-Schiff (PAS) and diastase periodic Acid-Schiff (D-PAS); (2) a positive serum GM-CSF antibody test which indicated an elevated serum GM-CSF antibody level [9]. All patients signed an informed consent form. Proteinaceous material positive for PAS staining and D-PAS staining was obtained from broncho-alveolar lavage fluid (BALF) or transbronchial lung biopsy (TBLB) or surgical lung biopsy (e.g. Video-assisted Thoracoscopic Surgery). The GM-CSF antibody test was performed according to the method established by Uchida et al. [10]. The cut-off point set by our center is 4 μg/ml, and measurements above this value are considered positive for the serum GM-CSF antibody test [11].

### Clinical data collection

All aPAP patients were patients in the China PAP registry study, who had a PAP-related condition assessment between November 15, 2021 and November 15, 2022, and were recorded into the China PAP registry study database. Patients were evaluated for demographics (including age, gender, smoking history, chronic medical history, previous treatment history, vaccination status), arterial blood gas (ABG) analysis, serology (including lactate dehydrogenase, carcinoembryonic antigen levels, cytokeratin 19 fragment antigen21-1 levels), pulmonary function test results, chest CT score, six-minute walk test (6MWD), St. George's Respiratory Questionnaire (SGRQ), and disease severity score (DSS). The DSS categories were defined by Inoue et al. as follows: Grade 1: No symptoms and an arterial oxygen partial pressure (PaO_2_) ≥ 70 mmHg; Grade 2: PaO_2_ ≥ 70 mmHg with symptoms; Grade 3: PaO_2_ between 60 and 70 mmHg; Grade 4: PaO_2_ between 50 and 60 mmHg; and Grade 5: PaO_2_ below 50 mmHg [12].

A post-COVID-19 infection health status questionnaire and telephone follow-up had been administered to all registered patients included in our center through the Xingshulin MedClip app. Both the questionnaire distribution and telephone follow-up had been completed between January 1, 2023 and February 15, 2023. The questionnaire had been presented as attachments (Additional file 1). Patients were only diagnosed with COVID-19 infection if they tested positive for SARS-CoV-2 nucleic acid or antigen test, and they were confirmed between November 15, 2022 and January 31, 2023. All studies and data collection were reviewed by the ethics committee of Peking Union Medical College Hospital (JS-2639). In this study, patients were considered to have decreased oxygen saturation only if the decrease in oxygen saturation at rest was greater than or equal to 3% from before [13].

### Statistical analysis

Continuous variables were reported as mean ± standard deviation or median (P25, P75), and categorical variables were reported as percentages N (%). We used the independent samples t-test or Mann–Whitney U test for continuous variables and the chi-square or Fisher exact probability test for categorical variables. Predictors of prognosis were evaluated using univariate analysis first to screen variables, then inconsistent variables were excluded according to collinearity, and finally multivariate logistic regression analysis was performed according to the representative variables selected in clinic. Two-sided test was performed, and *P* value < 0.05 was considered a statistically significant difference. All statistical analyses were completed using SPSS version 25.0 software and R version 4.2.0 software.

## Results

**Fig. 1:**
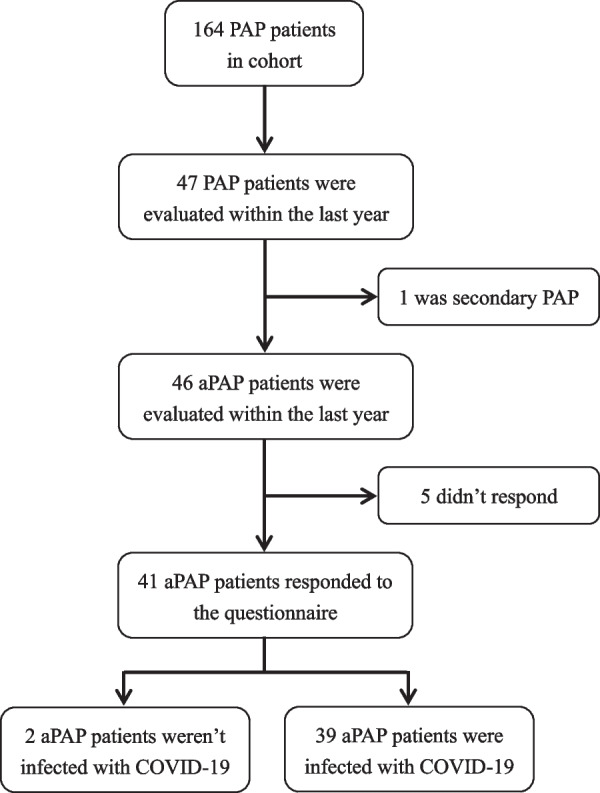
Flow diagram of the study cohort. *aPAP* Autoimmune pulmonary alveolar proteinosis

Ultimately, 41 patients with aPAP who had a PAP-related condition assessment within the last 1 year completed the questionnaire and telephone follow-up. Two patients were not infected with COVID-19, and the remaining 39 aPAP patients were infected with COVID-19 (Fig. 1). A total of 39 aPAP patients were included in this study. Twelve of the 39 patients (30.77%) had a decrease in oxygen saturation after COVID-19 infection.

### Baseline demographic information

The mean age of the 39 aPAP patients infected with COVID-19 was 42.56 (± 12.28) years; 25 out of 39 (64.1%) of them were male. Only 2 patients (5.1%) were diagnosed with PAP by Video-assisted Thoracoscopic Surgery (VATS), 14 out of 39 (35.9%) by BALF only, 4 out of 39 (10.3%) by TBLB only, and 19 out of 39 (48.7%) by both BALF and TBLB. All patients were positive for serum GM-CSF antibody test (> 4 μg/ml) with a median of 31.68 (19.31, 70.34) μg/ml. Nine out of 39 patients (23.1%) were ex-smokers and 8 out of 39 patients (20.5%) were current smokers. Eight out of 39 patients (20.5%) had been treated with whole lung lavage, 17 out of 39 (43.6%) had been treated with GM-CSF inhalation that is Molgramostim, and 10 out of 39 (25.6%) had oxygen therapy. Regarding the status of vaccination against the COVID-19, 11 out of 39 patients (28.2%) had never received the vaccine, 5 out of 39 (12.8%) had received 2 doses of the vaccine, and 23 out of 39 (59.0%) had received 3 doses of the vaccine (Table 1).

**Table 1:** Baseline demographic characters

Variables	All (n = 39)	Decreased oxygen saturation (n = 12)	Non decreased oxygen saturation (n = 27)	*P* value
Age (year)	42.56 ± 12.28	44.92 ± 14.70	41.52 ± 11.19	0.485
Male (%)	25 (64.1%)	6 (50.0%)	19 (70.4%)	0.287
BMI (kg/m^2^)	24.61 (22.51, 26.78)	23.42 (21.05, 26.67)	24.61 (22.64, 26.78)	0.480
Main symptoms lead to medical advice				0.203
Exertional dyspnea (%)	12 (30.8%)	5 (41.7%)	7 (25.9%)	
Cough (%)	16 (41.0%)	6 (50.0%)	10 (37.0%)	
Abnormal chest imaging found in physical examination (%)	11 (28.2%)	1 (8.3%)	10 (37.0%)	
Smoking status				0.719
Never (%)	22 (56.4%)	8 (66.7%)	14 (51.9%)	
Ex-smoker (%)	9 (23.1%)	2 (16.7%)	7 (25.9%)	
Current smoker (%)	8 (20.5%)	2 (16.7%)	6 (22.2%)	
History of tuberculosis (%)	5 (12.8%)	1 (8.3%)	4 (14.8%)	1.000
Hypertension (%)	5 (12.8%)	2 (16.7%)	3 (11.1%)	0.634
Diabetes (%)	3 (7.7%)	2 (16.7%)	1 (3.7%)	0.219
History of dust inhalation (%)	14 (35.9%)	4 (33.3%)	10 (37.0%)	1.000
History of oxygen therapy (%)	10 (25.6%)	6 (50.0%)	4 (14.8%)	0.043
History of systemic corticosteroids (%)	12 (30.8%)	3 (25.0%)	9 (33.3%)	0.719
History of WLL (%)	8 (20.5%)	5 (41.7%)	3 (11.1%)	0.079
History of GM-CSF inhalation (%)	17 (43.6%)	7 (58.3%)	10 (37.0%)	0.299
Coronavirus vaccine (%)				0.690
0	11 (28.2%)	4 (33.3%)	7 (25.9%)	
2	5 (12.8%)	2 (16.7%)	3 (11.1%)	
3	23 (59.0%)	6 (50.0%)	17 (63.0%)	

*BMI* Body mass index, *CT* Computer tomography, *GM-CSF* Granulocyte macrophage colony stimulating factor, *WLL* Whole lung lavage

We compared the 2 groups of patients with or without decreased oxygen saturation after COVID-19 infection and showed that patients who had previous oxygen therapy (decreased oxygen saturation vs. non decreased oxygen saturation: 6/12 vs. 4/27, *P* = 0.043) were more likely to have oxygen desaturation after COVID-19 infection. Patient age, gender, BMI, history of smoking, history of tuberculosis, comorbidities, history of whole lung lavage and GM-CSF inhalation therapy had no significant difference on whether patients had a decrease in oxygen saturation after COVID-19 infection. In addition, there was no significant difference in the number of doses of vaccination on whether oxygen saturation decreased after COVID-19 infection (*P* = 0.690) (Table 1).

### Effect of baseline laboratory test information on whether oxygen saturation decreased after infection with COVID-19

For baseline ABG, patients with lower baseline PaO_2_ were more likely to have decreased oxygen saturation after COVID-19 infection. PaO_2_ (decreased oxygen saturation vs. non decreased oxygen saturation): 74.50 ± 13.61 mmHg versus 86.49 ± 11.92 mmHg, *P* = 0.009. For other blood parameters, we also found that patients with higher baseline LDH levels were more likely to have decreased oxygen saturation. LDH (decreased oxygen saturation vs. non decreased oxygen saturation): 298 (234, 377) U/L versus 218 (197, 309) U/L, *P* = 0.037 (Table 2).

**Table 2:** Baseline laboratory test information

Variables	All (n = 39)	Decreased oxygen saturation (n = 12)	Non decreased oxygen saturation (n = 27)	*P* value
Arterial blood gas				
pH	7.41 ± 0.02	7.41 ± 0.03	7.41 ± 0.02	0.351
PaCO_2_ (mmHg)	37.56 ± 3.11	37.92 ± 2.35	37.40 ± 3.42	0.639
PaO_2_ (mmHg)	82.80 ± 13.50	74.50 ± 13.61	86.49 ± 11.92	0.009
SaO_2_ (%)	96.5 (95.3, 97.7)	95.4 (93.4, 97.7)	96.5 (95.7, 97.7)	0.245
A-aDO_2_ (mmHg)	21.8 (9.6, 31.2)	26.8 (16.0, 42.9)	21.8 (8.9, 27.7)	0.159
HCO_3_^−^ (mmol/L)	23.04 ± 1.85	23.48 ± 1.62	22.85 ± 1.94	0.339
Serology				
HGB (g/L)	158.36 ± 17.77	155.25 ± 21.39	159.74 ± 16.18	0.474
RBC# (× 10^12^/L)	5.21 ± 0.61	5.10 ± 0.82	5.26 ± 0.51	0.456
HCT (%)	45.65 ± 4.76	44.78 ± 6.00	46.04 ± 4.17	0.454
CEA (ng/ml)	4.00 (2.43, 7.60)	4.00 (2.29, 20.40)	3.90 (2.43, 7.30)	0.298
Cyfra21-1 (ng/ml)	4.60 (2.60, 9.50)	7.05 (4.00, 11.70)	3.60 (2.30, 7.52)	0.072
LDH (U/L)	243 (204, 350)	298 (234, 377)	218 (197, 309)	0.037
Pulmonary function				
FEV_1_ pred (%)	88.0 (75.5, 99.2)	90.0 (67.0, 104.7)	88.0 (77.0, 98.0)	0.799
FVC pred (%)	91.0 (77.0, 101.0)	86.0 (68.3, 105.5)	95.0 (78.0,101.0)	0.753
FEV_1_/FVC (%)	81.5 (77.9, 87.0)	83.6 (81.1, 87.8)	80.3 (76.8, 86.8)	0.221
TLC pred (%)	85.0 (73.0, 94.0)	83.9 (65.7, 103.7)	85.0 (77.0, 94.0)	0.799
RV pred (%)	86.0 (71.0, 99.0)	91.0 (73.6, 105.8)	85.0 (68.5, 99.0)	0.822
DLCO pred (%)	68.3 (52.1, 81.8)	63.3 (44.9, 83.8)	69.6 (63.2, 80.5)	0.284
DLCO/VA pred (%)	83.8 (75.4, 97.4)	77.0 (74.3, 93.6)	89.5 (78.2, 97.4)	0.036
6MWD (m)	512 (463, 570)	464 (406, 538)	532 (470, 575)	0.028
Borg at the end of 6MWD	1 (0, 2)	1 (0, 3)	0 (0, 2)	0.538
SGRQ				
Symptom	28 (12, 47)	43 (27, 69)	24 (9, 36)	0.010
Activity	29 (17, 47)	56 (23, 82)	23 (6, 36)	0.005
Impact	14 (1, 43)	40 (13, 56)	9 (0, 23)	0.022
Total	21 (9, 51)	46 (20, 64)	19 (8, 26)	0.009
DSS				0.017
1	13 (33.3%)	1 (8.3%)	12 (44.4%)	
2	20 (51.3%)	7 (58.3%)	13 (48.1%)	
≥ 3	6 (15.4%)	4 (33.3%)	2 (7.4%)	

*6MWD* 6 min walking distance (test), *A-aDO*_*2*_ Alveolar arterial oxygen gradient, *CEA* Carcinoembryonic antigen, *Cyfra21-1* Cytokeratin 19 fragment antigen21-1, *DLCO* Diffusing capacity for carbon monoxide, *DLCO/VA* Diffusing capacity for carbon monoxide corrected for alveolar volume, *DSS* Disease severity score, *FEV*_*1*_ Forced expiratory volume in the first second, *FVC* Forced vital capacity, *HCO*_*3*_^−^ Carbonic acid hydrogen radical, *HCT* Hematocrit, *HGB* Hemoglobin, *pH* Pondus hydrogenii, *LDH* Lactate dehydrogenase, *PaCO*_*2*_ Partial pressure of carbon dioxide, *PaO*_*2*_ Partial pressure of oxygen, *RBC* Red blood cell, *RV* Residual volume, *SaO*_*2*_ Oxygen saturation of blood, *SGRQ* St George Respiratory Questionnaire, *TLC* Total lung capacity

For baseline pulmonary function, DLCO/VA% (decreased oxygen saturation vs. non decreased oxygen saturation): 77.0 (74.3, 93.6) % versus 89.5 (78.2, 97.4) %, *P* = 0.036. Patients with lower DLCO/VA% were more likely to have decreased oxygen saturation after infection with COVID-19. However, the remaining baseline pulmonary function measures were not statistically significantly different in predicting whether patients had a decrease in oxygen saturation after infection with COVID-19 (Table 2).

Results from the baseline 6MWD and SGRQ were also both correlated with whether patients had decreased oxygen saturation after infection with COVID-19. 6MWD (decreased oxygen saturation vs. non decreased oxygen saturation): 464 (406, 538) m versus 532 (470, 575) m, *P* = 0.028; total SGRQ score (decreased oxygen saturation vs. non decreased oxygen saturation): 46 (20, 64) versus 19 (8, 26), *P* = 0.009 (Table 2).

The baseline DSS of PAP patients better predicted the likelihood that PAP patients would have decreased oxygen saturation after COVID-19 infection. Patients with higher DSS, which means who suffered more severe disease with PAP, who were more likely to have decreased oxygen saturation after COVID-19 infection (*P* = 0.017) (Table 2).

In univariate analysis, DSS, LDH level and DLCO/VA% were all predictors of whether oxygen saturation decreased after infection with COVID-19. However, in multivariate analysis, only the DSS categories was independent predictor of whether oxygen saturation decreased in patients infected with COVID-19 in this cohort (≥ 3; OR 24.000; 95% CI 1.689–340.992; *P* = 0.019).

### Symptoms and interventions after infection with COVID-19

After COVID-19 infection, patients may develop a variety of clinically relevant symptoms, the most frequent of which is fever, with 33 out of 39 patients (84.6%) experiencing fever after COVID-19 infection, followed by asthenia in 24 out of 39 (61.5%), expectoration in 20 out of 39 (51.3%), headache in 19 out of 39 (48.7%), pharyngalgia or cough in 18 out of 39 (46.2%), and a number of other COVID-19 relevant symptoms, including nasal congestion, rhinorrhea, hyposmia, hypogeusia, myalgia, and diarrhea, none of which correlated significantly with whether patients had decreased oxygen saturation. However, dyspnea symptoms were significantly correlated with decreased oxygen saturation (*P* < 0.001), with 7 out of 39 patients (17.9%) had new-onset dyspnea, 6 out of 39 patients (15.4%) had worsening dyspnea, and the remaining patients did not have dyspnea symptoms (Table 3).

**Table 3:** Symptoms and interventions after infection with COVID-19

Variables	All (n = 39)	Decreased oxygen saturation (n = 12)	Non decreased oxygen saturation (n = 27)	*P* value
Dyspnea				< 0.001
Never	26 (66.7%)	3 (25.0%)	23 (85.2%)	
New-onset	7 (17.9%)	5 (41.7%)	2 (7.4%)	
Worsen	6 (15.4%)	4 (33.3%)	2 (7.4%)	
Interventions				
Outpatient or emergency	13 (33.3%)	6 (50.0%)	7 (25.9%)	0.163
NSAIDs or analgesics	36 (92.3%)	11 (91.7%)	25 (92.6%)	1.000
Systemic corticosteroids	3 (7.7%)	3 (25.0%)	0 (0.0%)	0.024
Antibiotics	9 (23.1%)	6 (50.0%)	3 (11.1%)	0.014
Hospitalized	4 (10.3%)	4 (33.3%)	0 (0.0%)	0.006
GM-CSF inhalation				0.222
Never	26 (66.7%)	6 (50.0%)	20 (74.1%)	
Continuously used	8 (20.5%)	3 (25.0%)	5 (18.5%)	
Discontinue	5 (12.8%)	3 (25.0%)	2 (7.4%)	

*GM-CSF* Granulocyte macrophage colony stimulating factor

Thirteen out of 39 patients (33.3%) had outpatient or emergency department visits after COVID-19 infection; 4 out of 39 (10.3%) were hospitalized, all of whom had decreased oxygen saturation, but none were admitted to the ICU. Six out of 39 patients (15.4%) required additional oxygen therapy or had increased oxygen conditions than before, all of whom were treated with nasal catheter oxygen therapy. Thirty-six out of 39 patients (92.3%) were taking NSAIDs or analgesics, including but not limited to acetaminophen, ibuprofen, and loxoprofen sodium; 9 out of 39 (23.1%) were taking antibiotics; 3 out of 39 (7.7%) were on systemic corticosteroids. Moreover, only 2 patients took antivirals for COVID-19, one for Azvudine and another for Nirmatrelvir/ritonavir. Patients with decreased oxygen saturation after COVID-19 infection were more likely to require oxygen therapy (*P* < 0.001) and to be taking systemic corticosteroids (*P* = 0.024) and antibiotic (*P* = 0.014) medications. For aPAP patients on GM-CSF inhalation therapy, 8 out of 13 (61.5%) of such patients continuously GM-CSF inhalation therapy after COVID-19 infection compared with 5 out of 13 (38.5%) who discontinued it. There was no significant difference in whether aPAP patients continuously used or discontinued GM-CSF inhalation therapy after COVID-19 infection on whether patients had a decrease in oxygen saturation (Table 3).

## Discussion

In this study, we reported the clinical manifestations of patients with aPAP after infection with Omicron variants of SARS-CoV-2 for the first time. We found that aPAP patients with worse baseline respiratory status were more likely to have oxygen desaturation after COVID-19 infection, which means that such patients are more likely to have relatively serious symptoms such as dyspnea or worse prognosis after COVID-19 infection. In addition, we first reported the hospitalization rate of patients with aPAP after infection with Omicron strain, which was lower than the previously reported hospitalization rate of PAP patients infected with COVID-19 [5].

Our study found that the infection rate of COVID-19 in aPAP patients was a little bit higher than that estimated by the Chinese Center for Disease Control and Prevention (CDC) in the same period. The infection rate of COVID-19 in aPAP patients in this study was 95.12%, while the CDC estimated that the infection rate of COVID-19 in China was 82.4% as of February 7, 2023 [14]. We believe that this is likely due to the estimated results of the CDC through online survey. A considerable part of the population directly took non-steroidal anti-inflammatory drugs due to fever or other symptoms after COVID-19 infection, and did not report the infection, which may underestimate the infection rate of the COVID-19 pandemic in China. Meanwhile, a retrospective cohort study in Europe found that the infection rate of PAP patients was similar to that of the general population [5]. Therefore, we believe that aPAP does not increase the probability of COVID-19 infection in patients.

The main finding of this study was that patients with poor baseline respiration have a higher probability of hypoxia after COVID-19 infection. Previous research has not mentioned this point. Our study found that the indicators indicating the baseline respiratory status of patients with aPAP, previous oxygen therapy, PO_2_, DLCO/VA%, 6MWD, SGRQ total score, DSS categories, all of which showed that patients with poor baseline respiratory status were more likely to have oxygen saturation decline after COVID-19 infection. This is of great significance for the treatment of PAP patients and even patients with other interstitial lung diseases after infection with COVID-19. When patients with COVID-19 develop hypoxemia, it often means the progression of COVID-19, more serious symptoms, and worse prognosis. Therefore, for those PAP patients with poor baseline respiratory condition, when they are diagnosed with COVID-19 infection, doctors should pay more attention and take more active treatment strategies to avoid disease progression.

Another important finding was our study first reported the hospitalization rate of patients with aPAP infected with Omicron strain. Only 4 out of 39 patients were hospitalized, and the hospitalization rate was 10.3%, which is much lower than the previous reported in the European cohort with the rate of 35.5%. Meanwhile, all hospitalized patients in this study did not need ICU admission, and none of our patients including no responded patients died or need lung transplantation. However, almost 50% of hospitalized patients in the European cohort entered the ICU. Besides, there were also 2 out of 11 hospitalized patients died and 1 out of 11 hospitalized patients underwent lung transplantation [5]. This huge difference between the two studies may be caused by a variety of factors. Firstly, none of the patients in the European cohort study were vaccinated, while the vaccination rate of patients in our study was 71.8%. Although it was not found in our study that the dose of vaccination had a significant difference on whether patients would suffer from hypoxia after COVID-19 infection, a large number of previous studies have shown that vaccination can provide a very high level of protection and effectively reduce the rates of severity and mortality after COVID-19 infection [15]. Secondly, the SARS-CoV-2 strains infected by the patients in this study were all Omicron strains, while the European cohort did not mention what their infected strains were. Based on the enrollment time, it is speculated that the European cohort may be infected with Alpha or Delta strains, and Omicron strains should not be included. Different strains of the SARS-CoV-2 can lead to different infection and severity rates. Although the 10.3% hospitalization rate is not as high as expected, it is still relatively high compared to the general population (less than 1%), highlighting the vulnerability of the PAP population and the need for more attention after infection [16].

Our single-center study found that aPAP patients infected with COVID-19 in our cohort rarely used antiviral therapy. One out of 2 patients receiving antiviral therapy was hospitalized. Patients with high viral load of SARS-CoV-2 are more likely to develop severe disease, and early inhibition of viral replication can significantly improve the prognosis of patients with COVID-19 [17, 18]. PAP patients with worse baseline respiratory status are more prone to hypoxia after infection, so we’d like to suggest that PAP patients prescribe timely antiviral therapy.

GM-CSF plays a key role in host lung defense. The presence of anti GM-CSF antibodies in patients with aPAP leads to a reduction in the ability of alveolar macrophages to clear debris and pathogens, maintain surfactant homeostasis, and limit inflammation in the alveolar environment [19]. A study of inhaled GM-CSF in the treatment of COVID-19-related hypoxemia found that inhaled GM-CSF treatment could effectively improve A–aDO_2_ in patients [7]. The European cohort study found that previous inhalation of GM-CSF treatment had no effect on the outcome or hospitalization of PAP patients infected with COVID-19, which is consistent with our findings [5]. There was no significant difference in the decrease of blood oxygen saturation no matter whether GM-CSF inhalation therapy was used in the past (*P* = 0.299), or GM-CSF inhalation therapy was continuously used or discontinued after covid-19 infection (*P* = 0.222). Therefore, whether inhaled GM-CSF treatment has benefits for PAP patients with COVID-19 still needs to be explored.

As an interstitial lung disease, doctors should take different treatments and monitoring strategies for PAP patients infected with COVID-19 according to different conditions. An international multicenter study showed that patients with interstitial lung disease had higher mortality after infection with COVID-19 compared with patients without interstitial lung disease or other chronic lung diseases [4]. However, data on COVID-19 in rare lung diseases are still scarce. Although a European cohort study showed that PAP patients had higher hospitalization rate and mortality after infection [5], but there still lacks of guideline or standards for PAP patients to better cope with COVID-19 in the current medical system. Our research fills this gap well. PAP patients simultaneously infected with COVID-19 has the risk of aggravating COVID-19. For patients with mild PAP, the risk of progression of COVID-19 is not much. However, for patients with higher DSS and more severity of PAP, the probability of oxygen desaturation greatly increases. Vaccination, timely oxygen therapy, early use of antiviral medication, and timely vital sings monitoring may minimize the disease progression of patients. It’s urgent to establish a treatment strategy for patients with PAP after infected with COVID-19 in the future (Additional file 1).

There are some limitations in this study. Due to the fact that PAP is a rare lung disease and this study is a single center study, the number of PAP patients eligible for inclusion in the study is relatively small. In addition, due to the COVID-19 pandemic, many PAP patients have not been followed up in the past year due to the lockdown policy. This may lead us to underestimate the probability of serious events occurring in PAP patients infected with COVID-19. Although multivariate analysis was carried out, due to the limited number of patients and oxygen saturation decrease events, we only found DSS as a predictor to predict the possibility of patients' oxygen saturation decline. Whether PAP itself will progress after infection with COVID-19 in PAP patients is also a point of great interest to us, which requires further follow-up research.

## Conclusion

In conclusion, we found for the first time that the worse baseline respiratory status of patients with aPAP increased the probability of oxygen desaturation after infection with COVID-19. Meanwhile, we first reported the hospitalization rate of patients with aPAP caused by Omicron variant of SARS-CoV-2. All these will provide valuable data to make better medical strategies for PAP patients infected with COVID-19 in the future.

### Supplementary Information

**Additional file 1. **Questionnaire of pulmonary alveolar proteinosis patients infected with COVID-19.

## Data Availability

Raw data for Tables 1, 2 and 3 are not publicly available due individual privacy but are available from the corresponding author on reasonable request.

## References

[1] Trapnell BC, Nakata K, Bonella F, Campo I, Griese M, Hamilton J (2019). Pulmonary alveolar proteinosis. Nat Rev Dis Primers.

[2] Jouneau S, Ménard C, Lederlin M (2020). Pulmonary alveolar proteinosis. Respirology.

[3] McCarthy C, Carey BC, Trapnell BC (2022). Autoimmune pulmonary alveolar proteinosis. Am J Respir Crit Care Med.

[4] Drake TM, Docherty AB, Harrison EM, Quint JK, Adamali H, Agnew S (2020). Outcome of hospitalization for COVID-19 in patients with interstitial lung disease. An international multicenter study. Am J Respir Crit Care Med.

[5] Papiris SA, Campo I, Mariani F, Kallieri M, Kolilekas L, Papaioannou AI, et al. COVID-19 in patients with pulmonary alveolar proteinosis: a European multicentre study. ERJ Open Res. 2023;9(1). 10.1183/23120541.00199-2022 PMC9271262 36601310

[6] Powers KJ, Avadhanula V, Patel PR, Sarkar PK, Piedra P, Zarrin-Khameh N (2022). Whole lung lavage: treating pulmonary alveolar proteinosis at the time of COVID pandemic. Respir Med Case Rep.

[7] Paine R, Chasse R, Halstead ES, Nfonoyim J, Park DJ, Byun T (2022). Inhaled sargramostim (recombinant human granulocyte-macrophage colony-stimulating factor) for COVID-19-associated acute hypoxemia: results of the phase 2, randomized, open-label trial (iLeukPulm). Mil Med.

[8] Coirier V, Delamaire F, Chauvin P, Kerjouan M, Lederlin M, Maamar A (2023). A case report of Covid-19 in an autoimmune pulmonary alveolar proteinosis: an association in tune with the times!. Respir Med Case Rep.

[9] Tian X, Yang Y, Chen L, Sui X, Xu W, Li X (2020). Inhaled granulocyte-macrophage colony stimulating factor for mild-to-moderate autoimmune pulmonary alveolar proteinosis—a six month phase II randomized study with 24 months of follow-up. Orphanet J Rare Dis.

[10] Uchida K, Nakata K, Carey B, Chalk C, Suzuki T, Sakagami T (2014). Standardized serum GM-CSF autoantibody testing for the routine clinical diagnosis of autoimmune pulmonary alveolar proteinosis. J Immunol Methods.

[11] Li Y, Tian X, Gui Y, Ma A, Li X, Zeng N (2014). Serum markers in patients with idiopathic pulmonary alveolar proteinosis. Zhonghua Jie He He Hu Xi Za Zhi.

[12] Inoue Y, Trapnell BC, Tazawa R, Arai T, Takada T, Hizawa N (2008). Characteristics of a large cohort of patients with autoimmune pulmonary alveolar proteinosis in Japan. Am J Respir Crit Care Med.

[13] Nitzan M, Romem A, Koppel R (2014). Pulse oximetry: fundamentals and technology update. Med Devices (Auckl).

[14] Fu D, He G, Li H, Tan H, Ji X, Lin Z (2023). Effectiveness of COVID-19 vaccination against SARS-CoV-2 omicron variant infection and symptoms—China, December 2022–February 2023. China CDC Wkly.

[15] McMenamin ME, Nealon J, Lin Y, Wong JY, Cheung JK, Lau EHY (2022). Vaccine effectiveness of one, two, and three doses of BNT162b2 and CoronaVac against COVID-19 in Hong Kong: a population-based observational study. Lancet Infect Dis.

[16] Deng Y, Han S, Liu J, Guo L, Feng L, Liao Y (2023). The risks of death and hospitalizations associated with SARS-CoV-2 Omicron declined after lifting testing and quarantining measures. J Infect.

[17] Zheng S, Fan J, Yu F, Feng B, Lou B, Zou Q (2020). Viral load dynamics and disease severity in patients infected with SARS-CoV-2 in Zhejiang province, China, January–March 2020: retrospective cohort study. BMJ.

[18] Stankiewicz Karita HC, Dong TQ, Johnston C, Neuzil KM, Paasche-Orlow MK, Kissinger PJ (2022). Trajectory of viral RNA load among persons with incident SARS-CoV-2 G614 infection (Wuhan Strain) in association with COVID-19 symptom onset and severity. JAMA Netw Open.

[19] Wessendarp M, Watanabe-Chailland M, Liu S, Stankiewicz T, Ma Y, Kasam RK (2022). Role of GM-CSF in regulating metabolism and mitochondrial functions critical to macrophage proliferation. Mitochondrion.

