# Supplementary material for: Effects of COVID-19 infection in patients with autoimmune pulmonary alveolar proteinosis: a single-center study
Source: Orphanet J Rare Dis. 2023 Nov 11;18:353. doi: 10.1186/s13023-023-02950-9 (PMC10638736; doi:10.1186/s13023-023-02950-9)
Supplement: Supplementary file 1 — Additional file 1. Questionnaire of pulmonary alveolar proteinosis patients infected with COVID-19. [file 13023_2023_2950_MOESM1_ESM.docx]

Questionnaire of [pulmonary alveolar proteinosis](javascript:;) patients infected with COVID-19

1. Name:

Phone number:

2. COVID-19 vaccination status (multiple choice)

a. Unvaccinated

b. 1 dose, last vaccination time:

c. 2 dose, last vaccination time:

d. 3 dose, last vaccination time:

3. Have you had contact with a confirmed COVID-19 patient? Yes or No

4. Have you been diagnosed with COVID-19? Yes or No

5. If you are diagnosed with COVID-19, the way to confirm the diagnosis is

a. Nucleic acid test

b. Antigen test

c. No test

6. COVID -19 nucleic acid test or antigen test positive date:

7. Whether the nucleic acid test or antigen test turns negative? Yes or No

8. If your nucleic acid test or antigen test turns negative, then the date:

9. Have you experienced dyspnea or worsened after infection with COVID-19?

a. Never

b. New-onset

c. Worsen

10. What symptoms did you experience after infection with COVID-19? (multiple answers)

a. Nasal congestion

b. Rhinorrhea

c. Pharyngalgia

d. Headache

e. Hyposmia

f. Hypogeusia

g. Asthenia

h. Fever

i. Chill

j. Shiver

k. Myalgia

l. Arthralgia

m. Cough

n. Expectoration

o. Hemoptysis

p. Diarrhea

q. Nausea

r. Emesis

11. Did your oxygen desaturation after infection with COVID-19?

a. Yes

b. No

c. No test

Oxygen saturation before infection with COVID-19 (minimum value of no oxygen uptake in resting state):

Oxygen saturation after infection with COVID-19 (minimum value of no oxygen uptake in resting state):

12. Did you need extra oxygen after infection with COVID-19?

a. Need for oxygen, approximately the same as before infection.

b. Need for oxygen, increased time or increased oxygen flow.

c. No need for oxygen.

13. Oxygen intake method before infection with COVID-19.

a. Nasal catheter

b. [venturi mask](javascript:;)

c. High flow oxygen therapy

d. Other:

14. Oxygen intake method after infection with COVID-19.

a. Nasal catheter

b. [venturi mask](javascript:;)

c. High flow oxygen therapy

d. Others:

15. Have you discontinued GM-CSF (Sargramostim) inhalation due to infection with COVID-19?

a. Yes

b. No

c. Unused

16. Have you been seen in the emergency department or outpatient after infection with COVID-19? Yes or No

17. Medication after infection with COVID-19 (multiple answers):

a. NSAIDs (acetaminophen, ibuprofen, loxoprofen sodium etc.)

b. Systemic corticosteroids

c. Nirmatrelvir/ritonavir (Paxlovid)

d. Azulfidine

e. Antibiotics

f. Others:

18. Have you been hospitalized for infection with COVID-19? Yes or No

If yes, length of hospitalization:

19. Have you needed ICU admission if you hospitalized? Yes or No

If yes, length of ICU:

20. Have you used ventilator for assisted ventilation after infection with COVID-19?

a. Yes, [non-invasive ventilator](javascript:;).

b. Yes, invasive ventilator (intubation, tracheotomy).

c. Yes, [non-invasive ventilator](javascript:;) and invasive ventilator.

d. No
